# Novel Plasmid Transformation Method Mediated by Chrysotile, Sliding Friction, and Elastic Body Exposure

**Published:** 2007-02-14

**Authors:** Naoto Yoshida, Toshiaki Nakajima-Kambe, Kaori Matsuki, Toshiya Shigeno

**Affiliations:** 1 Department of Biochemistry and Applied Biosciences, University of Miyazaki, Miyazaki-shi, 889-2192, Japan.; 2 Graduate School of Life and Environmental Science, University of Tsukuba, Ibaraki, 305-8572, Japan.; 3 Takasaki Scientific Instruments Co., Saitama, 332-0021, Japan.

**Keywords:** chrysotile, elastic body, plasmid, sliding friction, transformation

## Abstract

*Escherichia coli* as a plasmid recipient cell was dispersed in a chrysotile colloidal solution, containing chrysotile adsorbed to plasmid DNA (chrysotile-plasmid cell mixture). Following this, the chrysotile-plasmid cell mixture was dropped onto the surface of an elastic body, such as agarose, and treated physically by sliding a polystyrene streak bar over the elastic body to create friction. Plasmid DNA was easily incorporated into *E. coli*, and antibiotic resistance was conferred by transformation. The transformation efficiency of *E. coli* cultured in solid medium was greater than that of *E. coli* cultured in broth. To obtain greater transformation efficiency, we attempted to determine optimal transformation conditions. The following conditions resulted in the greatest transformation efficiency: the recipient cell concentration within the chrysotile-plasmid cell mixture had an optical density greater than or equal to 2 at 550 nm, the vertical reaction force applied to the streak bar was greater than or equal to 40 g, and the rotation speed of the elastic body was greater than or equal to 34 rpm. Under these conditions, we observed a transformation efficiency of 10^7^ per μg plasmid DNA. The advantage of achieving bacterial transformation using the elastic body exposure method is that competent cell preparation of the recipient cell is not required. In addition to *E. coli*, other Gram negative bacteria are able to acquire plasmid DNA using the elastic body exposure method.

## Introduction

Discovering efficient means by which to introduce DNA into bacteria is of great practical importance in genetic engineering and molecular biology. The introduction of exogenous DNA into *Escherichia coli* was first demonstrated by Mandel and Higa (1970), who observed that incubation of a suspension of *E. coli* cells and bacteriophage λ DNA in a solution of CaCl_2_ at 0°C resulted in transformation. They further showed that exposure to a heat pulse, in which a mixture of cells and DNA was briefly incubated at 42°C, chilled on ice, and then diluted by the addition of growth medium, improved the frequency of transfection. Hanahan’s protocol is one of the best chemical transformation methods presently available, stemming from a series of attempts to attain maximal transformation efficiency (Hanahan, 1983; Inoue et al. 1990) after the first demonstration of Ca^2+^-dependent DNA transfer into *E. coli* by Cohen (1972; 1974).

Transformation of *E. coli* using high voltage electroporation was first described by Dower et al. (1988). *E. coli* can be transformed to extremely high efficiencies by subjecting a mixture of cells and DNA to brief but intense electrical fields of exponential decay waveforms (Antonov et al. 1993; Sheng et al. 1995). This process is highly dependent on two characteristics of the electrical pulse where as the electric field strength and pulse length. Shark et al. were the first to report physical transformation of a prokaryote (1991) using a simple and rapid method by which to introduce exogenous DNA into *Bacillus megaterium,* called the biolistic method. Using a biolistic propulsion system, they demonstrated acceleration of DNA-coated particles at high velocities into *B. megaterium* recipient cells. All strains of *B. megaterium* tested were successfully transformed using this method.

Appel et al. demonstrated that asbestos fibers, specifically chrysotile fibers, can act as transfection agents mediating the uptake of exogenous DNA into mammalian cells, such that genes of the exogenous DNA are subsequently expressed (1986). They also demonstrated that chrysotile fibers are at least as effective as calcium phosphate in standard transfection assays using optimal ratios of asbestos to DNA. Furthermore, following transfection with chrysotile asbestos, a minor percentage of plasmid DNA bearing a simian virus 40 origin of replication replicated 24 h after introduction.

We previously determined that chrysotile fibers penetrate the *E. coli* cell membrane, thereby generating sliding friction forces on agar plates, resulting in cell rupture (Yoshida and Saeki, 2004). We spread a mixture containing *E. coli* cells and chrysotile fibers onto 2% agar plates using a plastic stir stick. An apparatus was then developed to generate a sliding friction force on the surface of the plate by applying a fixed vertical reaction force to the stir stick while turning the plate. We referred to this as chrysotile-exposure. The number of living cells observed, expressed as colony forming units, was reduced in proportion to the duration of chrysotile-exposure. Transmission and scanning electron microscopy of the *E. coli* cells revealed chrysotile penetration of the cell membrane. The impact of exposing bacterial cells to chrysotile fibers was observed to depend more on physical processes and characteristics, such as penetration and size and shape, rather than chemical properties. The particulate nature of chrysotile fibers and their ability to bind nucleic acids has been exploited in transfection studies, in which chrysotile is used as a vehicle for introducing DNA into *E. coli* through cell interactions. We previously reported intracellular uptake of plasmid DNA by *E. coli* after spreading a suspension of recipient *E. coli* cells in the stationary phase, along with chrysotile and pUC18 donor DNA, over the surface of an LB agar plate and stirring several times with a stir stick (Yoshida et al. 2001). It was thus theorized that cell transformation by exogenous DNA was the result of penetration by chrysotile fibers to which DNA was adsorbed. In the present study, we systematically optimized various physical conditions and parameters with an aim to improve the efficiency of chrysotile-mediated transformation.

## Materials and Methods

### Cell culture

*Escherichia coli* strains, including JM109 (el4^−^, *rec*A1, *end*A1, *gyr*A96, *thi*-1, *hsd*R17, *sup*E44, *rel*A1, Δ(*lac-pro*AB), [F’, *tra*D36 *pro*AB, *lac*I^q^ ZΔM15]), were inoculated into Luria-Bertani (LB) agar plate medium or LB broth (Sambrook et al. 1989) and incubated or shaken for 15–18 h at 37°C.

### Preparation of the chrysotile colloidal solution

Chrysotile whisker, isolated using a sedimentation procedure (Pele and Calvert, 1983), was purchased from Waco Chemical Industries, Ltd. (Osaka, Japan). A 0.4 g whisker of chrysotile suspended in 40 ml of deionized distilled water was vigorously shaken for 5 min (at 1500 rpm), and then centrifuged at 2,000 *g* for 2 min. After centrifugation, the upper phase was discarded to remove un-required floating matter. The chrysotile pellet was then re-suspended in 40 ml of deionized distilled water, and vigorously shaken for 5 min (at 1500 rpm). The upper phase obtained by centrifugation at 2,000 g for 2 min was then filtered using a 30 μm mesh size nylon filter (Millipore, U.S.A.), autoclaved at 121°C for 15 min, and then used as chrysotile colloidal solution (CCS). Of the fibers remaining in the CCS, 95% had diameters of 50–70 nm and lengths of 0.34–2.30 μm, as determined by scanning electron microscopy. When chrysotile density was determined spectrophotometrically, an OD550 between 0.05 and 0.10 was observed, and the concentration of chrysotile fibers in CCS was estimated at 40–50 μg/ml.

### Preparation of the chrysotile-plasmid cell mixture

Bacterial colonies grown at 37°C for 18 hr on LB agar plates were retrieved and suspended in 475 μl of chrysotile colloidal solution containing 10–50 ng of plasmid DNA, such as pUC18 DNA, and subsequently added to 25 μl of 4 M NaCl solution as osmotic stabilizer. This was employed as the chrysotile-plasmid cell colloidal mixture (CPCM). After preparation of the CPCM including various concentrations of *E. coli* cells, *E. coli* cell density in CPCM was measured by absorbance at 550 nm.

### Standard protocol for elastic body exposure

The LB plates (8.5 cm in diameter), containing 2% agar and 50 μg/ml ampicillin, were rapidly dried in a clean room to remove all visible water from the surface before spreading of the chrysotile-plasmid cell colloidal mixture (CPCM) onto the plates. The moisture content of agar was 93% (w/w). A 50 μl aliquot of the CPCM was spread onto each LB plate, after which the surface of the plate was rubbed for 60 seconds using a stir stick (SARSTEDT, Germany) made with polystyrene using an automatic turning table, T-Au (various rotation speeds, ranging from 10–120 rpm) (Iuchi Co., Osaka, Japan), without damaging the agar. The vertical reaction force on the stir stick was maintained at 20–100 g using specially designed apparatus Tribos Provider (Yoshida and Saeki, 2004) for elastic body exposure. The Tribos Provider is purchased as Gene Injector from Takasaki Scientific Instruments Co. After incubation for 18–20 h at 37°C, the number of cell colonies demonstrating antibiotic resistance were counted. Transformation efficiency was expressed as the number of transformants per microgram of plasmid DNA.

## Results and Discussion

### Elastic body exposure

**Figure 1:**
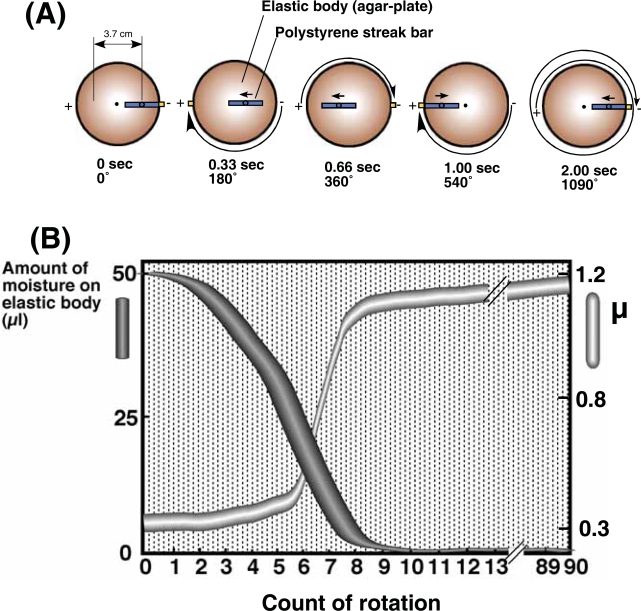
View demonstrating the isokinetics of the Tribos Provider apparatus (**A**). Relationship between reduction in surface moisture of agar streaked with the chrysotile-plasmid cell mixture and an increased coefficient (μ) of friction at the interface between the streak bar and agar with increased count of rotation (**B**). The elastic body rotated at 90 rpm. The width of the each curve represents the mean value ± SD of four independent experiments.

A colloidal mixture containing chrysotile, plasmid, and bacterial cells was spread onto the surface of an elastic body, such as agarose, and sliding friction applied to the surface of the elastic body using a polystyrene streak bar with a stably maintained vertical reaction force. The Tribos Provider apparatus (Yoshida and Saeki, 2004) can be used to apply sliding friction to the elastic body. We have termed this process elastic body exposure. Fig.1(A) demonstrates the isokinetics of the Tribos Provider, with the elastic body rotating at 90 rpm and the polystyrene streak bar applying a vertical force to an area of contact of 3.7 cm. The polystyrene streak bar moves 1.23 cm and 2.47 cm in the plus (+) direction while the elastic body rotates at an angle of 180 degrees for 0.33 seconds, and 360 degrees for 0.66 seconds, respectively. The bar reaches a distance of 3.7 cm after 1.00 second, and returns to its original position after 2.00 seconds. The elastic body rotates approximately three turns (1090 degrees) for each point of contact with the bar. These settings enable application of sliding friction across the whole surface of the elastic body, while regularly varying the direction of the sliding friction force. Fig. 1(B) shows the relationship between alterations in the aqueous volume of the chrysotile-plasmid cell mixture spread on the elastic body (the agar surface) and increased coefficients (μ) of sliding friction at the interface between the streak bar and agar. Initially, the agar absorbed moisture, but became dry after eight or nine turns. The sliding friction force generated is represented by the following formula: *F* = μ*W*, where *F*, μ, and *W* represent the sliding friction force, a coefficient, and the vertical reaction force, respectively (Gong et al. 1997; 1999; 2000). The frictional coefficient increased with increased rotations of the elastic body, since the vertical reaction force was fixed at 40 g. The coefficient was minimal after the first few rotations since moisture as lubricant still existed on the surface of the elastic body (agar). When the elastic body surface dried after eight or nine turns, both the coefficient and sliding friction force increased rapidly. The frictional coefficient remained high after nine turns of elastic body.

### Effect of cell density within the chrysotile-plasmid cell mixture on transformation efficiency

**Figure 2:**
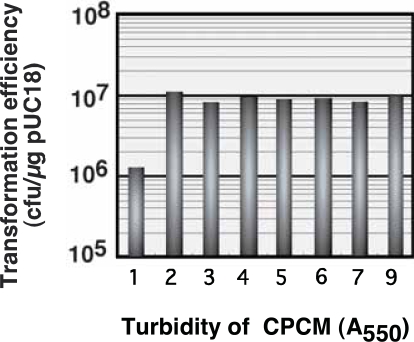
Optimal *E. coli* recipient cell concentration for efficient elastic body exposure-mediated plasmid (pUC18) transfection. Cell concentrations were determined based on the turbidity of the chrysotile-plasmid cell mixture (CPCM) at 550 nm. Each value represents the mean of three independent experiments. The error range was 2.1–6.2% of averages.

The chrysotile-plasmid (pUC18) mixture containing various concentrations of *E. coli* cells was subjected to elastic body exposure, using a vertical reaction force and elastic body rotation speed of 40 g and 90 rpm, respectively. The relationship between transformation efficiency and different cell densities of the chrysotile-plasmid cell mixture (CPCM) is shown in Fig. 2. Enhanced transformation efficiency by nearly seven orders of magnitude was observed using chrysotile-plasmid cell mixtures with optical density readings of 2 to 9 at 550 nm. In all experiments, few colonies demonstrating ampicillin-resistance were obtained at cell concentrations demonstrating an optical density reading of less than or equal to 1 at 550 nm. Thus, a recipient cell concentration within the chrysotile-plasmid cell mixture resulting in an optical density greater than or equal to 2 at 550 nm enhances transformation efficiency by 10-fold. Transformation efficiency was markedly affected by cell density in the CPCM, but reached a plateau at an optical density of 2 at 550 nm. This experiment had reliable reproducibility and demonstrated that *E. coli* cell density within CPCM plays an important role in *E. coli* transformation mediated by elastic body exposure.

### Effect of vertical reaction force on transformation efficiency

**Figure 3:**
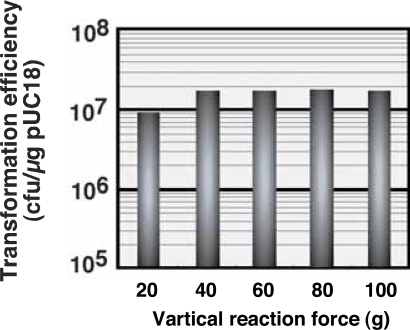
Optimal vertical reaction force for efficient elastic body exposure-mediated plasmid transfection. The vertical reaction force was adjusted using a Tribos Provider apparatus. A maximum vertical reaction force of 100 g was established, because more than 100 g of vertical reaction force was likely to destroy the elastic body. The area of contact between the streak bar and the elastic body was 1cm^2^. Each value represents the mean of three independent experiments. The error range was 1.6–5.2% of averages.

The effect of vertical reaction force on the poly-styrene streak bar on transformation efficiency at a fixed rotation speed of 90 rpm and an absorbance of 2 at 550 nm is shown in Fig. 3. Use of 40 g or more of vertical force doubled the transformation efficiency, compared to 20 g of force. The transformation efficiency decreased extremely, when use of less than 20 g of force. These results indicate that vertical reaction force on the agar medium is also an important parameter in the transformation of *E. coli* by elastic body exposure.

### Effect of rotation speed on transformation efficiency

**Figure 4:**
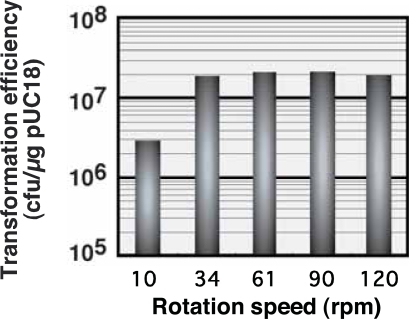
Optimal rotation speed of the elastic body for efficient plasmid transfection. Rotation speed was adjusted by adjusting the turn-table speed of the Tribos Provider apparatus. The area of contact between the streak bar and elastic body was 1cm^2^. Each point of contact by the streak bar took 2 seconds. The error range was 1.6–8.4% of averages.

The effect of rotation speed on transformation efficiency was investigated by fixing the optical density of CPCM at 550 nm and the vertical reaction force at 2 and 40 g, respectively. CPCM was subjected to elastic body exposure for 60 seconds on an LB plate containing 2% agar. As shown in Fig. 4, transformation efficiency was enhanced by more than seven orders of magnitude per microgram of pUC18 using a rotation speed of 34 rpm, compared to transformation at lower rpms. In contrast, a marked reduction in transformation efficiency was observed when the rotation speed was reduced to 10 rpm, resulting in less than 10^7^ transformants per microgram of pUC18. Transformation by elastic body exposure demonstrated high efficiency at rotation speeds exceeding 30 rpm.

The needle-like structure of chrysotile is thin enough to penetrate bacterial cells. Chrysotile aggregates are thought to penetrate the cell membrane of bacterial cells, thereby enabling bacterial cells to acquire plasmid DNA. Sliding friction results in penetration of bacteria by chrysotile, such as that generated between the agar surface and polystyrene streak bar. Thus, the cell density, the vertical force, and the rotation speed influences transformation efficiency.

### Effect of cell culture conditions on transformation efficiency

Recipient *E. coli* JM109 cells were cultured in LB broth or on LB plates at 37°C for 18 hr. *E. coli* cells from LB broth were harvested by centrifugation and dissolved in chrysotile colloidal solution containing pUC18 DNA and 200 mM NaCl. *E. coli* cells from the LB plates were picked up and added directly to the chrysotile colloidal solution. The respective chrysotile-plasmid cell mixtures were adjusted to an OD550 of 1.0 and subjected to elastic body exposure with vertical reaction force and rotation speeds of 40 g and 90 rpm, respectively. The transformation efficiencies of *E. coli* cells cultured in LB broth or on LB plates were compared, yielding 10^6^ and 5–6 × 10^6^ transfectants per microgram of pUC18, respectively. Thus, greater transformation efficiency is obtained using cells from a LB plate than from a LB broth. This suggests that cell wall properties are affected by broth or plate culture, thus, flexibility of the cell wall might be important in preventing cellular bursting following penetration by chrysotile (Yoshida and Saeki, 2004).

The present method was successfully applied to *E. coli* strains DH5α and C600, with transformation efficiencies of 1.3 × 10^6^ and 1.8 × 10^6^ per μg of pUC18 DNA, respectively. We determined that *E. coli* JM109 could also be transformed by plasmids or transposon genes to demonstrate kanamycin resistance using the elastic body exposure method, with transformation efficiencies of 1.1 × 10^5^ and 3.0 × 10^4^ per 1 μg of pHSG298 (Takeshita et al. 1987) and EZ::TN<KAN> transposon (Goryshin et al. 2000), respectively. Moreover *E. coli* can be transformed to demonstrate chloramphenicol resistance using this method, with a transformation efficiency of 1.0 × 10^5^ of pHSG396 (Takeshita et al. 1987). Transformation mediated by chrysotile fibers has been successful in *E. coli* strains, as well as other Gram negative bacteria, including *Pseudomonas* sp. and *Klebsiella aerogenes* (Azakami et al. 1992; 1995), before now. Therefore, this novel method will be beneficial for simple genetic manipulation, including genetic recombination, in bacteria. Chrysotiles have been studied in mutation tests using auxotrophic strains of *E. coli* and *Salmonella typhimurium* (Chamberlain and Tarmy, 1977). They reported that no mutagenic activity was found to be associated with any of chrysotiles tested over a wide range of concentrations.

Thus, we here describe *E. coli* transformation with plasmid using an elastic body exposure method. In brief, bacterial colonies cultured on a LB plate at 37°C for 18 h can be suspended in 475 μl of chrysotile colloidal solution containing 10–50 ng of plasmid DNA (pUC18), after which 25 μl of a 4M NaCl solution is added to the solution (the chrysotile-plasmid cell mixture; CPCM). The OD550 of the CPCM should be 2.0 or greater. The surface of the LB plates (8.5 cm in diameter) containing 2.0% agar and 50 μg/ml ampicillin should be rapidly dried in a clean room to remove all visible water before spreading of the CPCM. A 50 μl aliquot of CPCM should be spread onto the LB plate, and the surface aggravated for 60 seconds using a polystyrene streak bar and automatic turning table, without damaging the agar. Rapid drying on the surface of the plate upon exposure of CPCM to the elastic body (2% agar) is essential for transformation using the elastic body exposure method.

A great deal of effort has gone into the development of various transformation technologies, resulting in a large body of knowledge and diverse methodologies. Some methods are very simple but not widely applicable. Other methods are more useful but more complex or difficult to perform. Our method is simple, reliable, and highly reproducible, with the added beneficial that transformation and plating of the medium is performed simultaneously.
